# DiaReport: reproducible workflow for differential expression analysis and interactive reporting in DIA-based proteomics

**DOI:** 10.1093/bioinformatics/btag435

**Published:** 2026-06-22

**Authors:** Andrea Argentini, Esperanza Fernández, Jarne Pauwels, Kris Gevaert

**Affiliations:** VIB-UGent Center for Medical Biotechnology, VIB, Ghent B9052, Belgium; Department of Biomolecular Medicine, Ghent University, Ghent B9052, Belgium; VIB-UGent Center for Medical Biotechnology, VIB, Ghent B9052, Belgium; Department of Biomolecular Medicine, Ghent University, Ghent B9052, Belgium; VIB-UGent Center for Medical Biotechnology, VIB, Ghent B9052, Belgium; Department of Biomolecular Medicine, Ghent University, Ghent B9052, Belgium; VIB-UGent Center for Medical Biotechnology, VIB, Ghent B9052, Belgium; Department of Biomolecular Medicine, Ghent University, Ghent B9052, Belgium

## Abstract

**Motivation:**

Data-independent acquisition (DIA) has become the preferred data acquisition method for mass spectrometry-based proteomics, yet, reproducible workflows for differential expression (DE) analysis and results reporting remain limited. We present DiaReport, an R package that performs precursor- and protein-level DE analysis from DIA-NN output using MSqRob and QFeatures, while generating high-quality, interactive HTML reports through Quarto. DiaReport integrates precursor data, filtering of missing values, normalization, protein summarization and statistical modeling within a single function, supporting both simple pairwise as well as complex experimental designs. The package provides structured outputs and configuration files to ensure computational reproducibility across different studies. To accommodate diverse research needs, DiaReport includes multiple reporting templates tailored to different proteomic applications. Applying DiaReport to an extracellular vesicle (EV) proteomics dataset demonstrates its ability to efficiently analyze DIA data and provide rapid insights into sample quality and protein level differences.

**Availability:**

DiaReport is an open-source R package available at https://github.com/Gevaert-Lab/diareport (DOI: 10.5281/zenodo.20120604). The package is platform-independent and distributed under the MIT license. Reports are generated using Quarto and require only standard R dependencies. Detailed documentation, installation guides and usage vignettes are provided within the repository. The interactive HTML reports discussed in this study, including the UPS2 benchmark and EV case study, are archived on Zenodo (10.5281/zenodo.20122506 and 10.5281/zenodo.20123378).

## 1 Introduction

In mass spectrometry-based proteomics, data-independent acquisition (DIA) (Ludwig *et al.* 2018, Kitata *et al.* 2023) is rapidly gaining prominence over data-dependent acquisition (DDA). Unlike DDA, DIA systematically fragments all peptide ions within a defined mass-over-charge range, leading to a higher number of identifications and enabling more robust quantification. Among the available open-source tools for peptide-to-spectrum-matching, DIA-NN (Demichev *et al.* 2020) has emerged as a standard tool, supported by frequent updates and an active user community (Staes *et al.* 2024).

Differential expression (DE) analysis is a routine step in quantitative proteomics, supported by R packages such as MSstats (Kohler, Staniak *et al.* 2023, Kohler, Tsai *et al.* 2023), limma (Ritchie *et al.* 2015) and MSqRob (Goeminne *et al.* 2016, Sticker *et al.* 2020, Demeulemeester *et al.* 2024, Vandenbulcke *et al.* 2025). The MSstats ecosystem provides workflows for Tandem Mass Tag (TMT) labeling, Label-Free Quantification (LFQ), post-translational modifications (PTMs) (Kohler, Tsai *et al.* 2023) and Limited Proteolysis coupled to Mass Spectrometry (LiP-MS) (Malinovska *et al.* 2023), and supports multiple data sources, including DIA-NN output. MSqRob, paired with QFeatures (Gatto and Vanderaa n.d.), provides a flexible framework capable of handling complex experimental designs, as demonstrated in PTM (Demeulemeester *et al.* 2024) and TMT workflows (Vandenbulcke *et al.* 2025). However, despite the availability of strong individual components (e.g. DIA-NN for quantification and MSqRob for modelling), few tools integrate these steps into a reproducible and automated end-to-end workflow with high-quality reporting. Reporting is a critical component of data analysis, enabling users to explore results interactively and contextualize biological findings. Current efforts to bridge this gap include prolfquapp (Wolski *et al.* 2025) and MS-DAP (Koopmans *et al.* 2023). Prolfquapp is a command-line tool that generates differential expression analysis reports based on the prolfqua package (Wolski *et al.* 2023), utilizing a dedicated R Shiny application for downstream interactive visualization. MS-DAP offers an all-in-one R-based solution to generate static PDF reports. While MS-DAP supports a broader range of search engines (e.g. FragPipe (Kong *et al.* 2017), Spectronaut (Muntel *et al.* 2015), and MaxQuant (Tyanova *et al.* 2016)) and includes QC reporting, DiaReport provides an interactive alternative by utilizing framework such as Quarto (Allaire *et al.* 2022) to produce self-contained, portable HTML reports with Plotly-based visualizations (Plotly Technologies Inc. 2015) and dynamic tables (Xie *et al.* n.d.). Unlike manual GUI platforms like MSstatsShiny (Kohler, Kaza *et al.* 2023), DiaReport is a programmatic all-in-one R package designed for integration into automated high-throughput pipelines such as Nextflow (Di Tommaso *et al.* 2017) or Snakemake (Köster and Rahmann 2012).

While interactive reporting tools exist for quality control in MS-based proteomics (Olivella *et al.* 2021, Devreese *et al*. 2025,  Yue *et al.* 2026), equivalent frameworks for DE analysis remain limited. Computational reproducibility, another essential aspect of proteomic workflows, requires transparent documentation of processing steps, consistent software environments and standardized output formats (Wilkinson *et al.* 2025). Existing R-based proteomic workflows often rely on ad hoc scripts or loosely structured objects, making analyses difficult to reproduce while limiting interoperability with downstream tools. As DIA datasets grow in scale and complexity, such limitations increasingly hinder routine quantitative proteomics.

Here, we present DiaReport, an R package that performs precursor- and protein-level DE analysis of DIA-NN output using MSqRob and generates fully interactive HTML reports for DE analysis powered by Quarto. DiaReport provides a single entry-point function to execute the entire workflow, automatically applies the selected analysis template and stores results and configuration files in a structured directory format. This design enhances reproducibility and was created to simplify collaboration and facilitate deployment within research groups with limited programming or IT expertise.

## 2 Implementations

**Figure 1:**
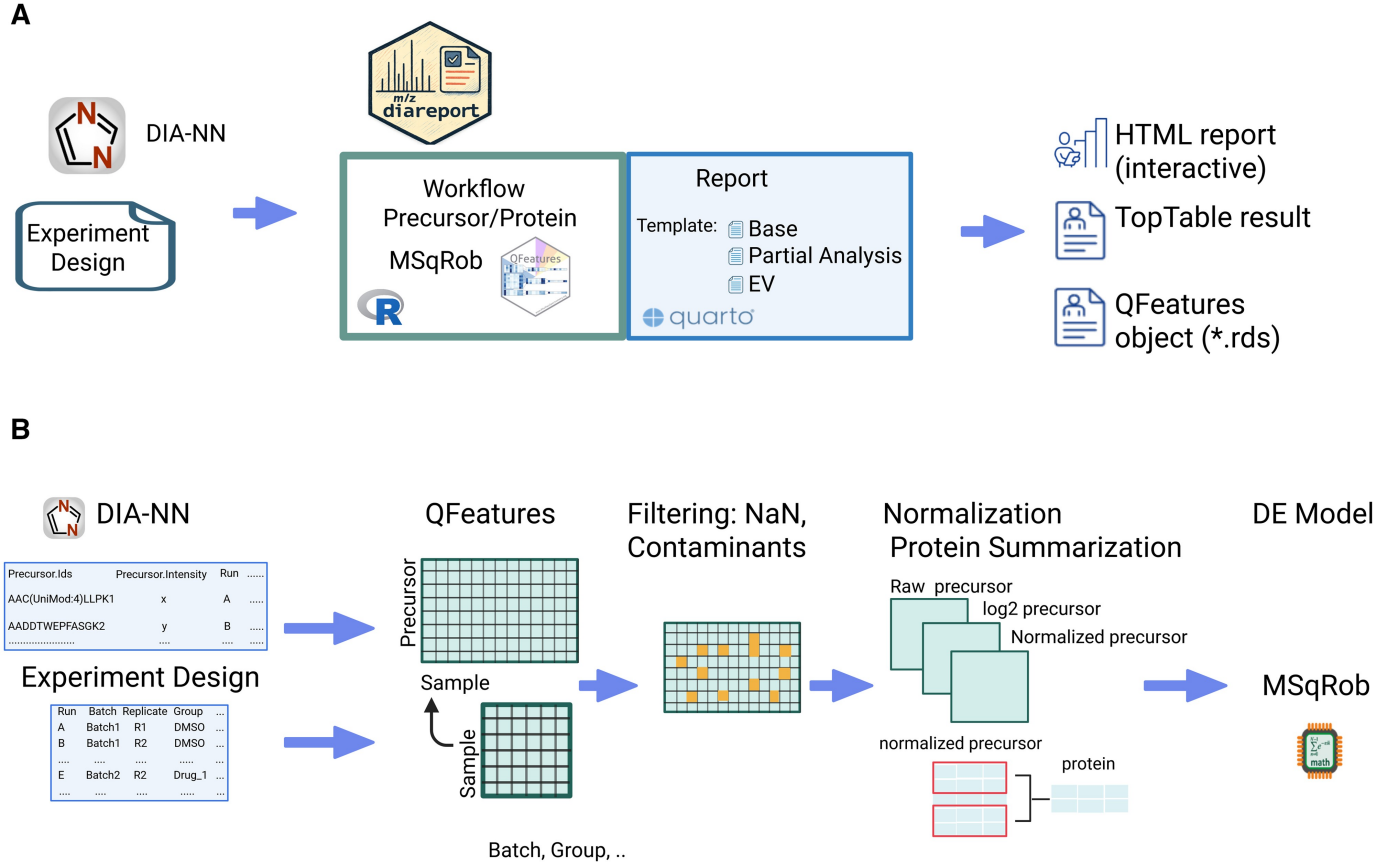
Overview of the DiaReport package and workflow. (A) Package architecture: DiaReport streamlines DIA-NN data processing using MSqRob and QFeatures, generating structured outputs including interactive HTML reports, top table results and a serialized QFeatures object for downstream analysis. (B) Precursor-level data and experimental metadata are integrated into a QFeatures object. The pipeline automates filtering (missing values, contaminants), log_2_-transformation and normalization before precursor-to-protein summarization and statistical modeling with MSqRob.

The DiaReport R package consists of two main components: (i) a data-processing module that prepares DIA-NN quantitative features for differential expression analysis and (ii) a reporting module that generates an interactive HTML report using Quarto templates (Fig. 1A). Both precursor- and protein-level workflows are supported within a single unified function interface.

### 2.1 DIA-NN output and experiment design file (EDF)

DiaReport requires two input files: DIA-NN report output (a parquet file or a tsv file for DIA-NN version < 1.8) and an experiment design file (EDF). The latter is a comma-separated table that specifies sample metadata (sample name, raw file, replicate and experimental group). Optional variables, including covariates and confounders, can be incorporated into the statistical model.

### 2.2 Data processing and differential analysis

The workflow follows a sequential pipeline: (i) precursor-level q-value and contaminant filtering, (ii) completeness-based filtering using the user-defined k threshold, (iii) log2-transformation and normalization and (iv) robust protein-level summarization (Fig. 1B).

Precursors that pass the fixed DIA-NN precursor- and protein-level *q*-value thresholds (*q* ≤ 0.01) are used to populate a QFeatures object. If matching-between-runs is enabled, filtering utilizes ‘Lib.PG.Q.value’ and ‘Lib.Q.value’ instead of global counterparts. Users can select either ‘Precursor.Quantity’ or ‘Precursor.Normalised’ for quantitative analysis.

As MSqRob explicitly models missing values, imputation strategies are currently not applied. To address the inherent prevalence of missing values in proteomics, DiaReport implements three completeness-based filtering strategies, defined by a user-specified completeness threshold k (expressed as a percentage): per-group completeness requires at least *k* percent valid values within at least one experimental group; across-groups completeness requires at least *k* percent valid values across all experimental groups simultaneously; and global completeness requires at least k percent valid values across the entire dataset, regardless of group assignment. These strategies allow users to balance the inclusion of condition-specific proteins (per-group) against the need for high-confidence features with minimal missingness (across-groups/global). A recommendation for the use of these filters is added in Table S1. Additional filters remove contaminants, restrict analysis to proteotypic precursors and ensure a user-defined minimum number of (different) precursors per protein. Intensities are log2-transformed and normalized using user-selected methods. The normalization methods allowed are the ones included in the QFeatures and MSqRob package, like quantiles, quantiles robust, difference of the median, and vsn. In the protein-level workflow, precursor intensities can be summarized using median polish or a robust summarization approach provided by MSqRob (Fig. 1B).

Differential expression analysis is performed using MSqRob at either the precursor or protein level. The user specifies the experimental design via an R formula argument (e.g. ∼ condition + batch), allowing for the flexible incorporation of multiple covariates or blocking factors.

### 2.3 Reporting layer and template

The reporting layer renders interactive HTML reports using Quarto templates. The architecture maintains a strict separation between data analysis logic and visualization, allowing for modularity and future expansion through custom templates. Currently, DiaReport includes three templates designed to meet increasing levels of analytical depth: the base template essentially provides quality control and differential expression analysis; the partial template adds ‘absent-from-DE’ analysis to identify features exclusively detected in specific condition groups; and the EV template offers domain-specific workflows for extracellular vesicle research, including marker panel summaries and contaminant interference analysis, which can be adapted to other biological contexts by supplying custom marker panels. While all templates support pairwise comparisons, the base workflow provides the greatest flexibility, enabling more complex experimental designs such as factorial models (Fig. 1B).

To ensure a clean and reproducible environment, processed results are saved as an RDS file and rendered from a temporary directory. All templates share a core set of features, including missing-value diagnostics, completeness summaries and PCA plots. Within the report, each comparison is organized into dedicated tabs containing interactive volcano plots, heatmaps and searchable result tables. The partial and EV templates further extend these capabilities by highlighting condition-specific features, with the EV template providing specialized visualizations for EV-specific quality metrics.

### 2.4 Structured output

In addition to differential expression analysis and interactive reporting, DiaReport enhances computational reproducibility by storing all analysis parameters in a YAML configuration file and improves result interoperability by saving all analytical outputs within a QFeatures object. Moreover, for each comparison, all plots generated in the report are exported in PDF format and the corresponding top-table results are saved as CSV files within a standardized, structured directory layout. These structured outputs, together with the QFeatures data objects, enable seamless integration with downstream tools, including R Shiny applications for interactive visualization of gene enrichment analyses.

## 3 Results

### 3.1 Base template analysis

DiaReport was validated using a UPS2/yeast benchmark (DIA-NN v1.8.1). The pipeline accurately recovered expected fold-changes for non-extreme concentration ratios (log_2_FC 1 and 2), achieving high sensitivity (TPR > 0.80) alongside minimal false-positive rates across all comparisons (Fig. S1 A-B, available as supplementary data at *Bioinformatics* online). Interactive PCA and UpSet plots further confirmed distinct sample clustering and high identification overlap across conditions (see report).

### 3.2 EV template analysis

To demonstrate DiaReport’s utility on a dataset extending beyond conventional quantitative benchmark studies, the tool was further evaluated on an in-house generated proteomics dataset comparing two different EV enrichment protocols: ultracentrifugation (UC) and an ultrafiltration strategy using 96-well plates (UF96) (Pauwels *et al.* 2025). Mass spectrometry data were processed using DIA-NN v2.2.0. The EV report generated by DiaReport was specifically tailored to gain rapid and comprehensive insights into the EV proteomics data by plotting several quality metrics, such as the abundance of several EV-specific protein markers (e.g. CD63 and CD81) and interference of contaminants at the precursor intensity level. In addition to EV-specific sections, the report summarizes key analytical outcomes, including the number of features retained after filtering, the effects of normalization and data completeness at the precursor- and protein-level across samples and conditions. Following data processing, 20 553 precursors were retained after filtering, corresponding to 2269 unique proteins. DiaReport analysis revealed that UF96 samples contained 40% fewer bovine contaminant precursors compared to UC samples, in line with previous findings. This was accompanied by a lower variability in EV protein marker abundance across UF96 replicates. Interactive PCA plots revealed a clear separation between the two isolation methods: UF96 triplicates formed a tight, well-defined cluster, whereas UC triplicates showed increased variability, with one replicate deviating from the remaining three. Differential protein analysis comparing UF96 and UC identified 425 proteins with significantly different abundance (FDR < 0.05, ∣log_2_FC∣>1), as represented in the report’s interactive volcano plot with accompanying tables. Among these, 128 transmembrane proteins were significantly up-regulated and 19 were down-regulated in the UF96 approach. Furthermore, 70 proteins were uniquely identified in the UF96 samples, while 168 were exclusive to UC, as listed in the “absent-from-DE” section of the report.

DiaReport provides the essential infrastructure to move from raw DIA-NN output to biological interpretation within a single, reproducible framework. For typical DIA clinical proteomics cohorts (50–200 samples), both the statistical analysis and report generation are complete within practical runtimes on a standard laptop (Table S2, available as supplementary data at *Bioinformatics* online). While the current implementation focuses on DIA-NN and MSqRob, the modular design facilitates future extensions to additional quantitative workflows and downstream analyses.

## Supplementary Material

btag435_Supplementary_Data

## Data Availability

The data underlying this article are available at the DiaReport Github, at https://github.com/Gevaert-Lab/diareport
