# Supplementary material for: DiaReport: reproducible workflow for differential expression analysis and interactive reporting in DIA-based proteomics
Source: Bioinformatics. 2026 Jun 22;42(7):btag435. doi: 10.1093/bioinformatics/btag435 (PMC13348718; doi:10.1093/bioinformatics/btag435)
Supplement: btag435_Supplementary_Data [file btag435_supplementary_data.zip › Supplementary_information_V1.pdf]

Supplementary Information

Strategy	Description	Recommended Use Case	Typical Study Type
**At least one condition**	Protein/precursor must be present in > k% replicates of at least one experimental group.	Captures true biological "on/off" switches (e.g., a protein entirely silenced in the control group but highly abundant in the disease group).	Clinical proteomics, discovery biomarker, medium-to-large biological studies
**Global**	Protein/precursor must be present in > k% replicates across the entire study, regardless of group.	Standard, general filtering. It assumes missing values occur completely at random due to technical drops rather than biological silencing	Controlled cell-line experiments, pilot studies, or initial feasibility runs where groups are highly homogeneous
**All conditions**	Protein/precursor must be present in > k% replicates of every experimental group	Highly stringent. Use when you cannot tolerate asymmetric missingness or when downstream statistical modeling requires a completely balanced matrix.	Clinical biomarker validation or high-stringency diagnostic studies.

Supplementary Table S1: Guidance for Missing Value Filtering Strategies in DiaReport.

This table provides a decision-making framework for selecting the optimal precursor/protein filtering strategy based on experimental design and sample complexity. Strategies range from inclusive approaches for pilot studies to high-stringency configurations for biomarker validation. The parameter k**%** represents the user-defined percentage of non-missing values required within the specified group(s) to retain a feature for downstream statistical analysis (e.g., k=70 requires a feature to be present in at least 70% of the replicates).

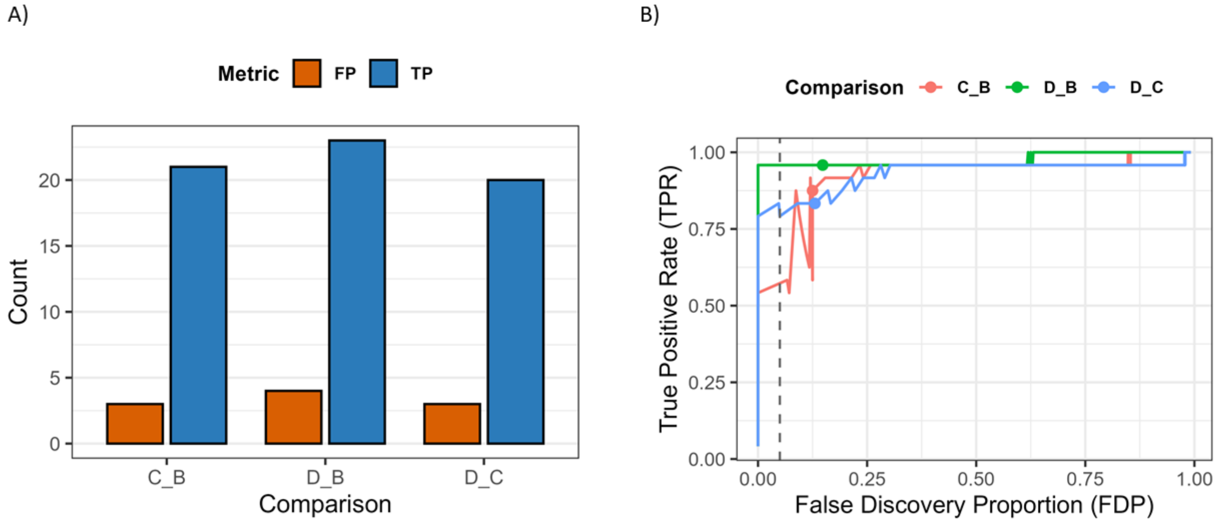

Supplementary Figure S1. Benchmarking Performance of DiaReport.

Performance was evaluated using median normalization and median polish summarization for three non-extreme concentration ratios (log_2FC of 1 and 2).

A) Absolute counts of True Positives (TP) and False Positives (FP). The bar plots represent the absolute counts of True Positives (TP) and False Positives (FP) identified by DiaReport at a 5% FDR threshold for each UPS2 spike-in comparison. Across all comparisons, the workflow consistently identified a high number of the expected spike-in proteins as True Positives, with constant False Positive occurrences (always below 5). This quantitative distribution validates the accuracy of the automated integration of DIA-NN quantification and MSqRob statistical modeling.

B) TPR versus FDP curves. The plot shows the True Positive Rate (TPR) versus the False Discovery Proportion (FDP for the comparisons C-B, D-B, and D-C. Each colored line represents a specific concentration comparison, with dots indicating the performance at a 5% False Discovery Rate (FDR) threshold. The vertical dashed line represents the nominal 5% FDP level, demonstrating that the MSqRob modeling within DiaReport maintains robust FDR control while achieving a high sensitivity (TPR) near 1.0 for most comparisons.
